# First Description of Infection of Caprine Herpesvirus 1 (CpHV-1) in Goats in Mainland France

**DOI:** 10.3390/pathogens5010017

**Published:** 2016-02-06

**Authors:** Florence Suavet, Jean-Luc Champion, Luc Bartolini, Maryline Bernou, Jean-Pierre Alzieu, Roland Brugidou, Séverine Darnatigues, Gaël Reynaud, Cécile Perrin, Gilbert Adam, Richard Thiéry, Véronique Duquesne

**Affiliations:** 1 Anses, Sophia-Antipolis Laboratory, 105 Route des Chappes, BP111, 06902 Sophia-Antipolis, France; florence_s@hotmail.com (F.S.); adamgilbert2003@yahoo.fr (G.A.); veronique.duquesne@anses.fr (V.D.); 2 Coopérative de l’Agneau de Haute-Provence, 04000 Digne Les Bains, France; jlucchampion@yahoo.fr; 3 Alpes-Maritimes Veterinary Laboratory, 06902 Sophia-Antipolis, France; lbartolini@cg06.fr; 4 Pyrénées-Tarbes Laboratory, Centre des Affaires Kennedy, 65000 Tarbes, France; m.bernou@labopl.com; 5 Ariège Veterinary Laboratory, Rue las Escoumes, 09008 Foix, France; jpalzieu@cg09.fr; 6 Aveyron Labo, 12031 Rodez, France; brugidou@aveyron-labo.fr; 7 Tarn Analytical Laboratory, 81011 Albi, France; severine.darnatigues@tarn.fr; 8 Savoie Analytical Laboratory, 73024 Chambéry, France; gael.reynaud@cg73.fr; 9 Anses, Niort Laboratory, 79012 Niort, France; cecile.perrin@anses.fr

**Keywords:** CpHV-1, caprine, Herpes, goat, epidemiology, France mainland

## Abstract

The purpose of this study was to investigate the epidemiological situation of the caprine herpesvirus 1 (CpHV-1) infection in nine districts in mainland France, mostly in the south, near Italy or Spain, where high seroprevalence has been observed. Two more central areas were also included in the study. The serosurvey was carried out in 9564 goats (275 herds) using bovine herpesvirus 1 (BoHV-1) glycoprotein B and E ELISAs. To confirm the presence of specific CpHV-1 antibodies, some of the samples were tested in neutralization assay. Results demonstrate, for the first time, CpHV-1 infection in goat herds on the French mainland. The analysis found cases of alphaherpesviruses infection in each district studied, with different levels of seroprevalence observed within each district (ranging from 0.2% to 31.56% at an individual level and from 9% to 46.2% for herd seroprevalence). Moreover, in the Alpes-Maritimes district, the seroprevalence seemed to be higher in older goats (79.45% of animals 6 years old or more) than in younger animals (40.99% of one-year-olds). This result suggests frequent virus re-excretion and circulation in herds. Results analysis also shows that the seroprevalence was higher when the herd size increased. In addition, the first French CpHV-1 strain was isolated from nasal swabs taken on an infected goat. The data reported herein demonstrate that CpHV-1 circulates in mainland France, which should henceforth be taken into consideration in cases of unexplained abortion in goats.

## 1. Introduction

Caprine herpesvirus 1 (CpHV-1) belongs to the subfamily of alphaherpesviruses, which contains seven genetically-related viruses [1]. CpHV-1 is closely related to bovine herpesvirus 1 (BoHV-1), responsible for infectious bovine rhinotracheitis (IBR) [2,3]. Like BoHV-1, CpHV-1 infects animals through the genital [4,5,6] or the respiratory mucosa [4] and establishes latent infection in sacral or trigeminal ganglia depending on the route of infection and the following spread through the body [5]. In natural infection, like most herpesviruses, it can reactivate in animals showing decreased immune status, generally as the result of stress (oestrus, transport, *etc.*) [6]. Although CpHV-1 infection is usually subclinical in adult goats, it can be responsible for different disorders including respiratory diseases, fever and leukopenia [7], vulvovaginitis [8,9] and balanoposthitis [10], and neonatal mortality. Abortions can be induced by the infection of pregnant goats at 3–4 months of gestation [11,12,13,14]. Severe disease may occur in neonatal kids characterized by pyrexia, conjunctivitis, oculonasal discharge, dyspnea, ulcerative and necrotic lesions throughout the enteric tract and high morbidity and mortality [15,16,17]. Abortions, stillbirths and reproductive failures cause financial losses in herds.

CpHV-1 infection was observed worldwide, e.g., in the USA [12,14,18], Canada [19] and New Zealand [20], usually linked with clinical features. In Europe, although the virus was first isolated in Switzerland [21], more recent serological studies suggested that infection is particularly located in Mediterranean countries such as Italy [22,23], Spain [24] and Greece [25]. In these countries, high seroprevalence is observed (between 21% in Spain and 60% in southern Italy). In France, which is geographically close to these countries, a first serological study was performed in the Mediterranean island of Corsica and in two central mainland districts [26]. The results showed a high seroprevalence in the southern part of Corsica (Corse-du-Sud district), but CpHV-1 infection was not identified in the few mainland districts studied [26,27].

To obtain a broader epidemiological view of the CpHV-1 status of goats in France, a serological survey was undertaken in nine districts in mainland France. The survey focused on districts located in southern regions, close to Italy or Spain and on two main goat-farming areas. The serological results show that CpHV-1 infection was detected in all districts studied. The influence of age and herd size on seroprevalence was examined. In addition, a CpHV-1 strain was isolated from seropositive goats.

## 2. Results

**Figure 1:**
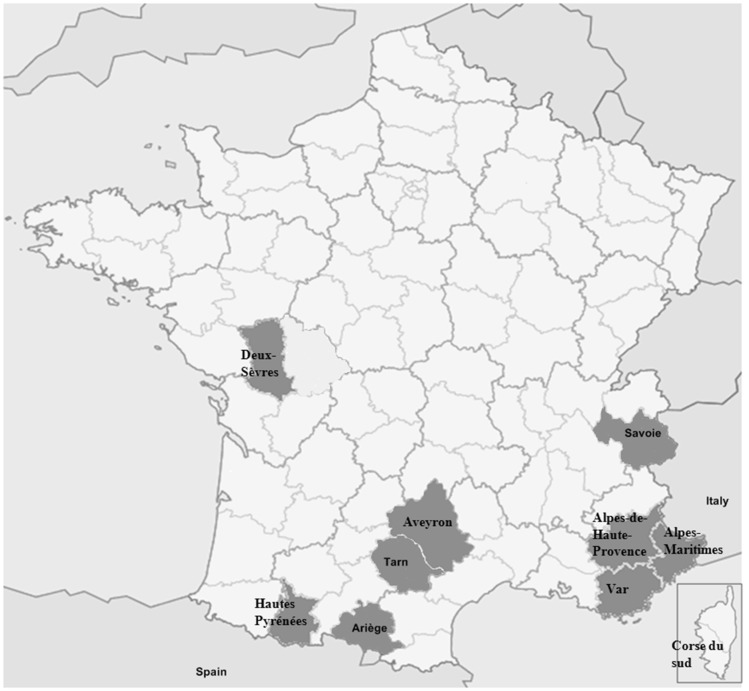
Map of France showing the location of districts studied. Grey shaded areas represent the nine districts of France in which the study was investigated: Alpes-Maritimes, Var, Alpes-de-Haute-Provence and Savoie districts (south east region) ; Ariège, Hautes-Pyrénées and Tarn districts (south west region); Deux-Sèvres and Aveyron districts (goat-farming region).

CpHV-1 infection was studied in several French districts in the south east, close to Italy (Alpes-Maritimes, Alpes-de-Haute-Provence, Savoie and Var), in the south west, close to Spain (Ariège, Hautes-Pyrénées and Tarn), and in two main goat farming areas (Deux-Sèvres in the Poitou-Charentes Region and Aveyron) (Figure 1). In all, 9564 goat serum samples from the nine districts were collected from 2006 to 2012.

**Figure 2:**
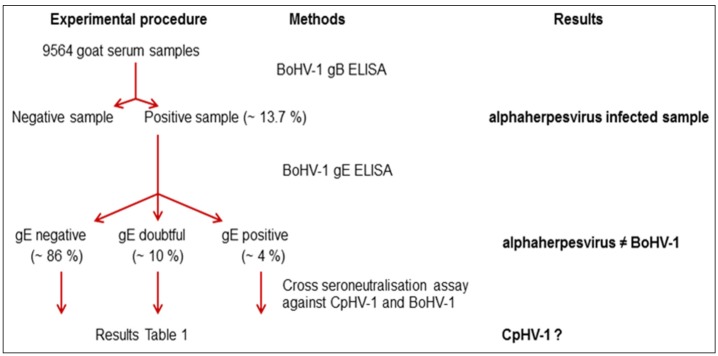
Experimental procedure used to analyse goat serum samples. The diagram represents the strategy of the herpes virus analysis in the study. A combination of glycoprotein B (gB) and glycoprotein E (gE) ELISA was used. All gB ELISA positive samples were analysed by a gE IBR ELISA. Cross neutralisation assay was performed on these samples to confirm the caprine herpes virus infection.

The serological status of the goats against CpHV-1 was obtained by using a combination of commercial bovine ELISA tests designed to detect gB and gE antibodies, and cross seroneutralisation assay against CpHV-1 and BoHV-1 (Figure 2). As shown previously [26], gB+/gE− reactivity is strongly indicative of CpHV-1 infection. Cross seroneutralisation tests were also undertaken to confirm the CpHV-1 status.

All gB positive sera were tested with the BoHV-1 gE ELISA commercial kit. Most sera were gE negative (86%), suggesting that goat herds were infected by an alphaherpesvirus related to BoHV-1 (Figure 2).

These results were confirmed by comparative virus neutralisation tests (VNT) against CpHV-1 or BoHV-1 (Table 1). The data obtained using serum samples from CpHV-1 experimentally-infected calves and BoHV-1 experimentally-infected calves demonstrates that the method possesses good specificity. Sera with negative results in gB and gE ELISAs were also negative in VNT. ELISA gB-positive goat sera preferentially neutralize CpHV-1 virus, whatever the gE ELISA serum profile. Therefore CpHV-1 virus seems to be responsible for alphaherpesvirus infection in French goat herds.

**Table 1:** Comparative viral neutralisation test performed against bovine herpesvirus 1 (BoHV-1) and caprine herpesvirus 1 (CpHV-1). Results shown are an example of results obtained using serum samples from a herd located in the Alpes-Maritimes district. Different profiles of serum were analysed.

Categories of Serum Samples	Neutralizing Antibody Titre Against	
	CpHV-1	BoHV-1
Calf experimentally infected with CpHV-1	>1/32	0
Calf experimentally infected with BoHV-1	1/2	>1/32
gB negative/gE negative	0 0	0 0
gB positive/gE negative	1/4 >1/32	1/1 1/4
gB positive/gE doubtful	>1/32 >1/32	1/2 1/1
gB positive/gE positive	>1/32 >1/32	1/4 1/2

Positive herds were found in all the districts studied, thus demonstrating goat CpHV-1 infection on the French mainland (Table 2). The seroprevalences at the individual and herd level varied greatly according to the geographical location. Herds from the southern districts close to the Italian or Spanish borders showed relatively high herd seroprevalence (up to 46.1% in Ariège), but the individual seroprevalence within herds also differed greatly in these regions (Table 2). The highest individual seroprevalences were found in the south-east (*i.e.*, Var and Alpes-Maritimes, 30% and 31.5% respectively).

It is worthy of note that despite the high level of seroprevalence at herd level (up to 46.1%) in the Pyrenean districts (Ariège and Hautes-Pyrénées) and Aveyron, individual apparent seroprevalence remains low (about 3%). In contrast, the individual seroprevalence in Deux-Sèvres, which is the main goat farming area, is only 0.2%. Overall, among 275 analyzed herds located in mainland France, 30% present a CpHV-1 infection for an individual apparent seroprevalence of 14%.

**Figure 3:**
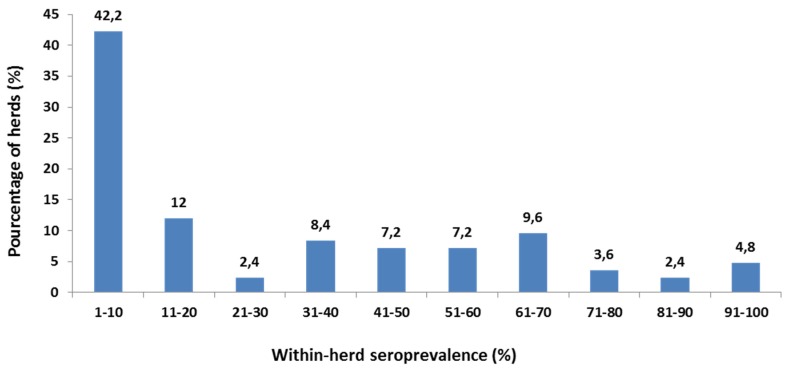
Distribution of the within-herd seroprevalence of CpHV-1 in 83 infected herds. The study comprised 275 goat herds and a total of 9564 animals. The percentage of seropositive animals was stratified into 10 categories. The histogram indicates the percentage of herds in each category.

The analysis of the distribution of within-herd prevalence of CpHV-1 in 83 infected herds shows that 42.2% of infected herds (n = 35) had a within-herd prevalence lower than 10% (Figure 3). In 27.7% of infected herds (n = 23) the total within-herd prevalence was more than 51%. Most of these herds are located in Alpes-Maritimes and Var.

**Table 2:** Goat serum samples gB ELISA screening. Based on the antigenic homology between CpHV-1 and BoHV-1 glycoproteins B (gB), ELISAs were performed using a commercial BoHV-1 gB ELISA (IDEXX laboratories).

Districts		Herd-level seroprevalence			Individual-level seroprevalence		
		Number of herds tested	Number of herds herpesvirus gB positive	Herd seroprevalence ^1^	Number of goats tested	Range of individual seroprevalence in infected herds	Mean individual apparent seroprevalence^1^
South-East districts	**Alpes-Maritimes**	114	39	**34.2%** (25.5%–42.9%)	3161	2%–100%	**30%** (28.3%–31.5%)
	**Alpes de Haute-Provence**	14	3	**21.4%** ^2^	356	6.2%–8.1%	2.2% (0.7%–3.8%)
	**Var**	18	5	**27.7%** (7.1%–48.4%)	697	20%–87.5%	**31.5%** (28.1%–35%)
	**Savoie**	20	7	**35%** (14.1%–55.9%)	555	0.6%–68.7%	7.2% (5.1%–9.4%)
South-West districts	**Hautes-Pyrénées**	18	4	**22.2%** (3%–41.4%)	289	3.8%–25%	4.1% (1.9%–6.5%)
	**Tarn**	11	1	9% ^2^	391	3.7%	0.5% (0.1%–1.2%)
	**Ariège**	39	18	**46.1%** (30.5%–61.8%)	2064	1%–18.2%	2.5% (1.9%–3.1%)
Main goat farming areas	**Aveyron**	21	4	**19.1%** (2.3%–35.9%)	1024	2%–52%	2.8% (1.8%–3.8%)
	**Deux-Sèvres**	20	2	10% ^2^	1027	2% ^2^	0.2% ^2^
	***Total***	**275**	**83**	**30.2%** (24.7%–35.6%)	**9564**		**13.7%** (13%–14.4%)

^1^ Seroprevalence expressed in percent with 95% confidence interval in brackets (exact binomial). ^2^ the confidence intervals were not evaluated because of the low studied populations.

**Figure 4:**
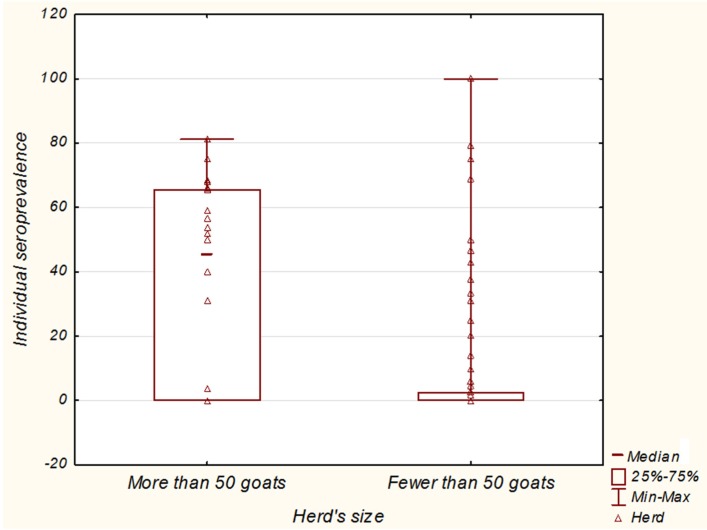
Analysis of the link between herd size and individual seroprevalence. Herds are sorted into 2 groups according to herd size. The box plot indicates the individual seroprevalence for each group.

In addition, the relationship between herd size and individual seroprevalence or infection status of herds was investigated. The 115 herds analyzed in Alpes-Maritimes were sorted into 3 groups depending on the herd size. In this district, most of the herds are small and many goat breeders own fewer than ten goats. There were 22 herds of 50 goats or more (1860 animals), 43 herds of from 10 to 50 goats (1088 animals) and 50 herds of fewer than 10 goats (213 animals). gB ELISA results corresponding to the 3161 goats tested were analyzed. The larger herds have significantly higher individual apparent seroprevalence than the other groups (Figure 4).

*p* < 0.005 (Non parametric Mann-Withney test).

**Figure 5:**
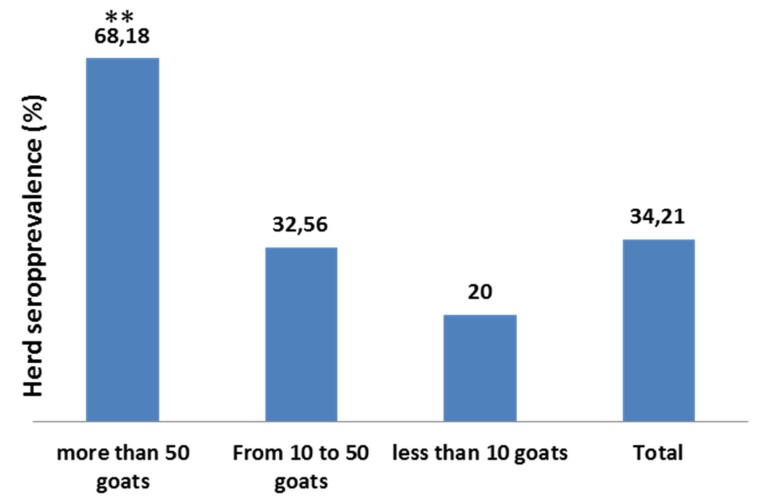
Analysis of the link between herd size and herd seroprevalence. Herds are sorted into 3 groups according to herd size. The histogram indicates the herd seroprevalence for each group (** *p* < 0.0005).

These large herds are also more frequently infected (Figure 5). Thus herd size was linked with both the serological status of the herd and with the individual seroprevalence in each herd.

**Figure 6:**
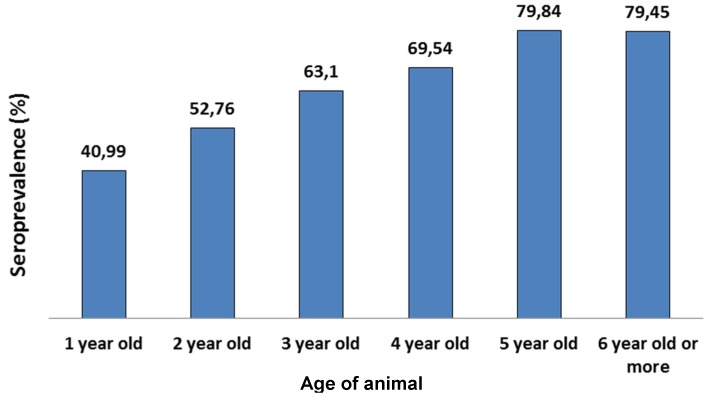
Analysis of seroprevalence according to age. Animals are sorted into 6 groups according to age. The histogram indicates the individual seroprevalence for each group.

The effect of the age of animals on individual seroprevalence was also studied. The analysis was done one 14 large herds located in Alpes-Maritimes (1281 animals). These animals were stratified according to their age into 6 different categories from one year to six years or more. The number of animals in each group ranged from 124 to 322 animals. Results show that seroprevalence increases with the animal’s age (Figure 6). Similar results were obtained for infected herds from Var (data not shown).

Viral isolation was performed to further confirm the CpHV-1 infection. To this end, 2 goat herds located in Var in which CpHV-1 antibodies were found in sera samples taken in 2008 were investigated again in 2011. Serum samples, nasal and genital swabs were taken from adult seropositive goats and kids. 18 goats from Herd A and 13 goats from Herd B were sampled. Interestingly, although apparent seroprevalences of Herds A and B were 70% in 2008, no significant change of seroprevalence was observed for Herd B in 2011 while seroprevalence of Herd A became null.

Viral strain culture was performed on MDBK cells from nasal or genital swabs**.** Viral multiplication was assessed by specific PCR for glycoprotein B and glycoprotein E.

**Figure 7:**
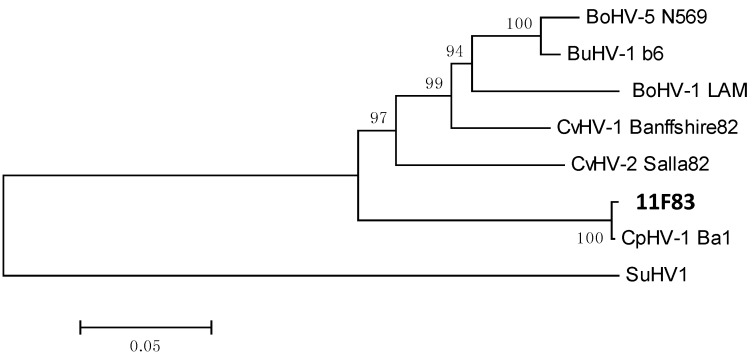
Neighbour-joining phylogenetic tree (933 bp) based on the combined gB and gE nucleotide sequences. The published sequences of alphaherpesvirus strains used in comparison are as follows: BoHV-5 N569 gB (AF078726.2), BoHV-5 N569 gE (EF624468.1), BoHV-1 LAM gB (EF624475.1), BoHV-1 LAM gE (EF624467.1), CvHV-1 Banffshire82 gB (EF624479.1), CvHV-1 Banffshire82 gE (EF624471.1), CvHV-2 Salla2 gB (AF078727.2), CvHV-2 Salla2 gE (EF624472.1), CpHV-1 Ba1 gB (EF624477.1), CpHV-1 Ba1 gE (EF624470.1), BuHV-1 b6 gB (EF624476.1), BuHV-1 b6 gE (EF624469.1) and SuHV-1 Becker (JF797219.1).The tree was rooted using SuHV-1 sequence as an outgroup. The scale bar represents 0.05 substitutions per site.

Weak band were obtained for genital swabs from Herd A, but most appeared negative by PCR after cell culture. However PCR products with the expected size (443 bp and 624 bp respectively) were obtained from nasal swabs taken from this herd after two rounds of amplification on MDBK cells. In addition, no amplification of viral CpHV-1 DNA from Herd B was obtained. PCR products obtained from gB and gE PCR were sequenced. The phylogenetic analysis obtained with compiled gB and gE gene regions is presented in Figure 7.

Sequences were compared with those from different ruminants alphaherpesviruses found in the literature. They display a strong homology with the CpHV-1 Ba1 strain, close to 100% (no difference between the French CpHV-1 gB sequence and the reference strain; 3 bases are different between the two strains for the gE sequence). Similar results were obtained when the phylogenetic analysis was performed separately with the 414 nt of the gB region or the 519 nt of the gE region. We were therefore able to confirm that we had successfully isolated a CpHV-1 strain from France, named 11F83.

## 3. Discussion

Our results demonstrate, for the first time, the presence of CpHV-1 infected goat herds on the French mainland. The current study presents the evidence of CpHV-1 infection occurring in mainland France, with an apparent seroprevalence of 13.73%. The highest rates of individual seroprevalence were found in the two Mediterranean districts studied, Alpes-Maritimes and Var. This is in agreement with previous reports describing CpHV-1 infection preferentially located in Mediterranean countries [22,23,24,25,26]. Moreover, in this area close to Italy, the four districts studied present herd seroprevalence greater than 20%. These observations are closely related to the considerable seroprevalence observed in Italy, even though Italian epidemiological studies were performed in Southern Italy [22,23].

In south-western districts, CpHV-1 individual seroprevalence is lower. Nevertheless, many herds contain one or several seropositive animals. In this region, about 27% of the herds tested showed CpHV-1 seropositive goats. Herd seroprevalence is especially high in the two districts located close to Spain. The two previous studies performed in Spain were done in Andalusia [24,27]. Individual seroprevalence was close to 22%, but these epidemiological studies were performed with a limited number of serum samples. It would be interesting to investigate the CpHV-1 seroprevalence in the Spanish Pyrenees, close to the French border.

Results obtained in Deux-Sèvres and Aveyron district showed that CpHV-1 infection also occurs in the main French goat farming areas, even if the prevalence of the infection is low. However, in Deux-Sèvres district, only 20 herds out of about 1200 herds listed were tested (1027 goats tested out of 160,500 goats listed). It would be interesting to perform a larger study in the entire Poitou-Charentes Region (which includes three other districts) where about 2500 herds are listed (about 270,000 goats).

Marked regional differences were observed. Differences in agricultural structure and geography can partially explain the regional variation of seroprevalence among goats. In the Alpine and Pyrenean regions, pasturing of goats is a traditional farming practice. Animals from many farms generally graze during summer on Alpine pastures where wild and different domestic ruminants frequently share grazing areas and water sources, which increases the risk of transmitting infectious disease agents, and then return in the autumn to their respective home farms. This proximity between ruminants during pasturing may partially explain the higher herd and individual seroprevalence in the South-East and also in the South-West region close to Spain. From this point, it will be interesting to continue the serological survey in France in the major goat farming areas and in neighbouring countries.

An analysis of within-herd seroprevalence shows that for more than 42% of infected herds, this was less than 10%. This result is consistent with the low individual seroprevalence observed in several districts. On the other hand, total within-herd prevalence was more than 51% in some herds located in Alpes-Maritimes, Var and also in Savoie and Aveyron districts. These results are consistent with the high individual seroprevalence in these districts.

Results analysis shows that both seroprevalence and herd infection status are correlated with herd size. Larger herds are more frequently infected and present significantly higher within-herd seroprevalence. The venereal route is considered the main route of transmission [5], in fact the frequency of infection in large herds could also be explained by the higher degree of commercial movement of latently infected animals between farms. The greater within-herd seroprevalence could also be explained by the higher density of animals in large herds, thus increasing contact between animals. Results also show that individual seroprevalence increases regularly with the animal’s age. This suggests virus re-excretion by infected animals in herds.

To isolate a CpHV-1 strain, samples from two herds (A and B) located in Var district were taken at two different times of year, first in 2008 and then over a second phase in 2011. Interestingly nasal swabs were positive whereas vaginal swabs were found negative by PCR. This surprising result may be related to the period of sampling. The results obtained for Herd A shows that serological status of herds and/or animals could change over time. This result is supported by other results obtained in Alpes-Maritimes district (data not shown). Specific antibody titres may not persist in goats for many years as was previously suggested [28]. As a consequence, the sampling period seems to be an important factor for accurately determining the infection status of a herd. Sequencing data showed that the isolated CpHV-1 strain is close to the only strain that have been sequenced to date.

## 4. Experimental Section

### 4.1. Cells and Virus

The Madin-Darby bovine kidney (MDBK) cell line (ATCC CCL-22) was maintained in Modified Eagle’s Medium (MEM) supplemented with 10% of foetal bovine serum (FBS) and glutamine (complete medium). The viral stocks of BoHV-1 Los Angeles strain (ATCC VR188) and CpHV-1 E/CH strain [2] were produced by infection of MDBK cells at a multiplicity of infection (MOI) of 0.5 in complete culture medium. When the cytopathic effect reached 90%, the cells underwent three cycles of freezing at −80 °C followed by thawing. The culture medium was then removed and clarified by centrifugation. Viruses were titrated by plaque assay on MDBK cells and used for comparative virus neutralization test (VNT).

### 4.2. Study Area and Samples

Goat serum samples were collected in several districts in south east France (Alpes-Maritimes, Var, Alpes-de-Haute-Provence and Savoie), south west France (Ariège, Hautes-Pyrénées and Tarn) and the main goat-farming areas (Deux-Sèvres and Aveyron district) (Figure 1). For all districts except Deux-Sèvres, the sera were collected in the context of the official monitoring required for the prophylaxis of brucellosis. As a consequence, there was no specific selection of herds (simple random sampling). In Alpes-Maritimes, Savoie, Tarn, Hautes-Pyrénées and Ariège districts, every animal in the selected herds was tested. Goat herd size ranged from 1 to 251 animals in Alpes-Maritimes, from 1 to 161 in Savoie, from 1 to 69 goats in Hautes-Pyrénées, from 17 to 53 goats in Tarn and from 22 to 128 goats in Ariège. In Aveyron, Var and Alpes-de-Haute-Provence districts, all animals in the herd were sampled only when fewer than 50 goats were present. Fifty serum samples selected randomly were tested when the herd size was larger. In Deux-Sèvres, on the other hand, goats were intentionally sampled from mixed herds (cattle and goats) for the purpose of this study. Fifty serum samples were tested from each of 20 large herds (from 110 to 1200 goats). Viral isolation was performed on nasal and vaginal swabs collected from animals from two positive herds located in Var district.

### 4.3. ELISA

The combination of two enzyme-linked immunosorbent assays (ELISA) based on the detection of BoHV-1 glycoproteins B (gB) and E (gE) was previously shown to be effective for distinguishing between BoHV-1 and related alphaherpesvirus infections in goats [26]. We therefore first screened goat sera for the presence of ruminant gB alphaherpesvirus-specific antibodies using a commercial blocking ELISA (HERDCHEK IBRgB, IDEXX laboratories, Switzerland) according to the manufacturer’s instructions. The interpretation of the percentage of competition was the same as is used for cattle (animals were considered positive when the optical density, expressed as a percentage, was above 45%). The gB-antibody-positive sera were then tested for BoHV-1 glycoprotein E (gE) specific antibodies by using a commercial blocking ELISA (BHV-1 gE antibody test kit, IDEXX laboratories, Westbrook, ME, USA. Animals were considered positive when the S/N (sample/negative control) ratio was less than or equal to 0.6 and considered as doubtful when the S/N value was greater than 0.6 but less than or equal to 0.7.

### 4.4. Comparative Virus Neutralisation Test

To further check the specificity of the antibody response, *i.e.*, to discriminate between CpHV-1 and BoHV-1 antibodies, a panel of sera showing different response profiles with respect to the two ELISAs was analysed in VNT. Several animals per profile were tested. After filtration, serum samples were heated to 59 °C for 30 min. Serial twofold dilutions of each serum sample tested were incubated in 96-well microplates at 37 °C for 24 h either in the presence of 100 DECP_50_ of BoHV-1 Los-Angeles strain or in the presence of 100 DECP_50_ of CpHV-1 E/CH strain. MDBK cells (2.0 × 10^5^ cells/mL) were then added to each well. Readings were made after incubation for 3 days at 37 °C. Viruses used in the running test were titrated (3 twofold dilutions) to verify the infectivity of the virus. The CpHV-1 titer was evaluated to 10^1,15^ TCID per well in the same time of the neutralizing test. The affinity of a serum with a herpesvirus strain was expressed as the reciproqual of the highest dilution of serum sample inhibiting cytopathic effect. Positive reference sera were obtained by experimental infection of calves infected intranasally with CpHV-1 E/CH strain or BoHV-1 Los Angeles strain.

### 4.5. Viral Isolation

Viral isolation was performed on nasal and vaginal swabs collected from animals from positive herds. Briefly, swabs were resuspended in 1 ml of sterile phosphate buffered saline (PBS) and frozen at −80 °C until use. The PBS suspension was inoculated into 24-well microplates containing an MDBK cell monolayer, grown in complete medium supplemented with antibiotics (penicillin, streptomycin). Cell monolayers were checked daily for cytopathic effects due to herpesvirus infection. When the first cytopathic effects appeared, supernatants were collected, centrifuged at 1500 rpm for 20 min and inoculated again into MDBK cells. When the cytopathic effect reached 50%, culture medium was removed, clarified by centrifugation, and analysed by conventional PCR.

### 4.6. PCR and Sequence Analysis

Total DNA was extracted from each cell culture supernatant using Dneasy Blood & Tissue kit (Qiagen, Courtaboeuf, France) according to the manufacturer’s instructions. Two different sets of consensus primers were used to amplify parts of glycoprotein B and glycoprotein E genes from different ruminant alphaherpesviruses [29,30]. The PCR and sequencing of a region of glycoprotein B gene (UL27, 443 bp) was performed using the primers CR30 (5'-TCGAARGCCGAGTACCTGCG-3' sense) and CR31 (5'-CCAGTCCCAGGCRACCGTCAC-3' antisense) [29]. The 624 bp region of the glycoprotein E gene was amplified using Alpha/US8/914F (5'-CGARACSTGCATCTTYCACC-3') and Alpha/US8/1538R (5'-GGSTCGTTGSTYGGM-3') [30]. PCR fragments were purified with QIAquick PCR Purification kit (Qiagen, Courtaboeuf, France). Direct sequencing of both strands of DNA was performed by Millegen (Labège, France). For both the gB (GenBank accession number KC846905) and the gE (GenBank accession number KC986906) regions of the genome, sequence alignments and phylogenetic trees were calculated with the CLUSTAL X (Version 1.81) analysis program [31]. An analysis of the compiled 933 nucleotides (nt) of gB and gE of the different alphaherpesviruses was also performed with the same method. The phylogenetic tree was constructed using the Neighbour-Joining method (Outgroup: SuHV-1). Phylogenetic and molecular evolutionary analyses were conducted using MEGA version 4 [32].

### 4.7. Statistical Analysis

The 95% confidence interval (95% CI) of the apparent prevalence was estimated using a binomial exact distribution. [33]. Statistical analyses were carried out using Statistica version 12 software. Chi^2^ test was used to compare the apparent prevalence between the different sizes of herds. The non-parametric Mann-Whitney test was used to compare the individual seroprevalence between the two different groups of herds. The limit of statistical significance of the conducted test was defined as *p* < 0.05.

## 5. Conclusions

This CpHV-1 infection in France could have a possible role in cases of unexplained abortions in several goat herds. This virus should also be taken in consideration since it may provoke severe genital lesions, respiratory disorders and neonatal mortality. The correlation between serological response and clinical features in herds (abortion) needs further study.

Finally, this study suggests that it would be interesting to investigate more widely the serological status of goat herds in France and neighbouring countries. In France particularly, investigations should focus on areas of intensive goat farming and the south of France.
